# The relationship between patients’ income and education and their access to pharmacological chronic pain management: A scoping review

**DOI:** 10.1080/24740527.2022.2104699

**Published:** 2022-09-01

**Authors:** Nicole Atkins, Karim Mukhida

**Affiliations:** Department of Anesthesiology, Pain Management and Perioperative Medicine, Dalhousie University, Halifax, Nova Scotia, Canada

**Keywords:** chronic pain, socioeconomic status, education, income, opioids, triptans, DMARDs

## Abstract

**Background:**

Though chronic pain is widespread, affecting about one-fifth of the world’s population, its impacts are disproportionately felt across the population according to socioeconomic determinants such as education and income. These factors also influence patients’ access to treatment, including pharmacological pain management.

**Aim:**

A scoping review was undertaken to better understand the association of socioeconomic factors with physicians’ pain management prescribing patterns for adults living with chronic pain.

**Methods:**

An electronic literature search was conducted using the EMBASE, CINAHL, SCOPUS, and Ovid MEDLINE databases and 31 retrieved articles deemed relevant for analyses were critically appraised.

**Results:**

The available evidence indicates that patients’ lower socioeconomic status is associated with a greater likelihood of being prescribed opioids to manage their chronic pain and a decreased likelihood of receiving prescription medications to manage migraines, rheumatoid arthritis, and osteoarthritis.

**Conclusions:**

These results suggest that individuals with lower socioeconomic status do not receive equal prescription medicine opportunities to manage their chronic pain conditions. This is influenced by a variety of intersecting variables, including access to care, the potential unaffordability of certain therapies, patients’ health literacy, and prescribing biases. Future research is needed to identify interventions to improve equity of access to therapies for patients with chronic pain living in lower socioeconomic situations as well as to explain the mechanism through which socioeconomic status affects chronic pain treatment choices by health care providers.

**Abbreviation:**

SES: socioeconomic status; RA: rheumatoid arthritis; IV: intravenous; SC: subcutaneous; bDMARDs: biological disease-modifying antirheumatic drugs; DMARDS; disease-modifying antirheumatic drugs; TNFi: tumour necrosis factor inhibitors; NSAIDs: non-steroidal anti-inflammatory drugs

## RÉSUMÉ

**Contexte**: Bien que la douleur chronique soit répandue, touchant environ un cinquième de la population mondiale, ses effets sont ressentis de manière disproportionnée au sein de la population en fonction de déterminants tels que l’éducation et le revenu. Ces facteurs influencent également l’accès des patients aux traitements, y compris la prise en charge pharmacologique de la douleur.

**Objectif**: Un examen de la portée a été entrepris pour mieux comprendre l’association entre les facteurs socioéconomiques et les tendances manifestées par les médecins en matière d’ordonnances pour la prise en charge de la douleur chez les adultes vivant avec une douleur chronique.

**Méthodes**: Une recherche documentaire électronique a été effectuée à l’aide des bases de données EMBASE, CINAHL, SCOPUS et Ovid MEDLINE. Par la suite, 31 articles récupérés qui avaient été jugés pertinents pour les analyses ont été soumis à une évaluation critique.

**Résultats**: Les données disponibles indiquent que le statut socioéconomique inférieur des patients est associé à une plus grande probabilité de se voir prescrire des opioïdes pour la prise en charge de leur douleur chronique et à une diminution de la probabilité de recevoir des médicaments sur ordonnance pour prendre en charge les migraines, la polyarthrite rhumatoïde et l’ostéoarthrite.

**Conclusions**: Ces résultats indiquent que les personnes ayant un statut socioéconomique inférieur ne bénéficient pas des mêmes possibilités de médicaments sur ordonnance pour prendre en charge leur douleur chronique. Cela est influencé par une variété de variables croisées, y compris l’accès aux soins, le fait que certains traitements soient potentiellement inabordables, la littératie de santé des patients et les préjugés en matière d’ordonnances. D’autres études sont nécessaires pour recenser les interventions visant à améliorer l’équité d’accès aux traitements pour les patients souffrant de douleur chronique vivant dans des situations socioéconomiques plus faibles, ainsi que pour expliquer le mécanisme par lequel le statut socioéconomique affecte les choix de traitement de la douleur chronique par les prestataires de soins de santé.

## Introduction

Recognized to be a disorder unto itself in the 11th revision of the *International Classification of Diseases*,^1^,^2^ chronic pain is “an unpleasant sensory and emotional experience associated with, or resembling that associated with, actual or potential tissue damage … that persists or recurs for longer than 3 months.”^3^ Chronic pain affects approximately one-fifth of the population and is a leading cause of disability and disease burden.^4,5^ Given that chronic pain is a complex and multidimensional experience whose assessment, study, and management are influenced by a variety of biopsychosocial contexts,^6^ it is not surprising that a variety of studies have found that patients’ socioeconomic status (SES) can affect their experiences, treatments, and health outcomes.^7–9^ Patients’ chronic pain experiences have been shown to be affected by factors such as their gender, ethnicity, social class, income, education, and the neighborhood in which they reside.^7,9–11^ The Canadian Pain Task Force^12^ agrees that such factors play a role in explaining the inconsistent and inequitable experience of pain and access to pain care in Canada. Therefore, among the six recommendations that the Canadian Pain Task Force has suggested to the Canadian federal government to improve the prevention and management of chronic pain are those specifically related to improving both equitable access to pain care and ensuring “equitable approaches for populations disproportionately impacted by pain.”^12(p1)^

A better understanding of the role of societal inequities in the development and severity of chronic pain is necessary in order to actualize such recommendations. Lower SES has been correlated with increased chronic pain incidence, severity, and pain-associated and psychosocial disability in a variety of studies performed using methodologies ranging from random population sampling to health questionnaires and surveys to prospective cohort studies following patients from birth to adulthood.^8,13–17^ In addition to being a risk factor for the development for disabling chronic pain,^18,19^ lower SES has been associated with a higher incidence of other medical comorbidities that can amplify chronic pain.^8,18^

Associations and interactions between lower SES and chronic pain are due in part to income-related factors. Income has been considered in different ways in chronic pain–related studies, including as measured household income, household wealth that incorporates the financial resources accumulated over a lifetime, employment type, indexes that include a combination of housing tenure, car ownership, and employment status, as well as self-reported ratings of economic hardship and daily financial worry.^8,14,17,20–22^ Studies report that living or growing up in a lower income household is associated with an increased risk of developing or reporting chronic pain and an increased prevalence and severity of chronic pain.^23–28^ As wealth increases, the degree to which chronic pain interferes with the performance of activities of daily living decreases.^20,29^ Self-perceived economic hardship has also been shown to exacerbate chronic pain.^22^ Income can affect health outcomes in terms of access to resources within health care, as well as resources outside of health care related to healthy living, such as access to healthy food and opportunities for physical activity.^20^ Lower-paying jobs may be associated with greater occupational hazards or be more physically demanding and so predispose to the development of chronic pain.^16,20,30^ For patients with lower income and chronic pain, this has meant decreased access to pain therapies or resources to modify their living or work environments to decrease their pain and/or improve their function.^20^

Closely linked to considerations of income and SES are those related to patients’ educational achievements. Education is associated with the acquisition of beliefs and knowledge that enables individuals to integrate health behaviors into their lifestyles and provides a sense of control over one’s own health.^31^ In health research, education is often measured by asking participants to identify the highest level of education that they have completed.^31,32^ Lower educational achievement has been associated with a higher prevalence of pain, more severe pain, and greater occupational and overall disability.^6,32–34^ Higher educational achievement was found to be a protective factor for developing chronic pain when controlling for occupational class and working conditions.^6,32^ A variety of hypotheses have been proposed to explain these relationships between lower levels of education and chronic pain. Higher levels of education can facilitate patients’ improved communication with physicians.^6,34^ Patients are therefore better able to access and understand treatment information, express themselves and their concerns, appropriately use care resources, and navigate health care systems more effectively.^6,34–37^ Physicians are more likely to spend more time with patients with higher SES, provide them with information, and justify treatment suggestions compared to patients with lower SES.^34,38–40^ Other studies suggest that lower levels of education are associated with fewer vocational options and occupations that require more physical work and thus workers are more prone to injuries and have less autonomy.^33,41–46^ Additionally, lower levels of education have been suggested to be a proxy for maladaptive pain beliefs and ineffective and passive coping strategies that exacerbate chronic pain.^6,32,47–50^ Roth and colleagues^6^ found that the belief that chronic pain is synonymous with “harm” and catastrophizing accounted for the association between lower educational achievement and severe disability secondary to chronic pain. They speculated that lower levels of education preclude patients’ abilities to develop accurate models of health and illness such that they develop ineffective ways to manage their pain, such as catastrophizing, praying, and hoping.^6,32,47^

There is evidence available to suggest that these aforementioned components of patients’ SES can have an association with physician medication prescription patterns. The writing of prescriptions is the most frequent medical intervention performed by physicians and is an integral component of the health care system.^51^ Therefore, understanding the factors that influence prescribing patterns is important for assessing the quality of care provided to patients. However, though previous studies have evaluated the influence of guidelines on the pharmacological management of chronic pain, the association of patients’ SES on such patterns is not well understood.^52–55^ The aim of this study was to better identify this knowledge gap by surveying the available literature, examining how research on this topic has been conducted, and providing an overview of its focus and the available evidence. The aim was not to produce a “critically appraised and synthesized result to answer a particular question,” which would be in keeping with a systematic review.^56(p3)^ Thus, a scoping review was deemed to be the most appropriate method of accomplishing these aims.^56^ Examination of this topic has been suggested to be important given the impetus for chronic pain to be considered a pressing public health problem and increased recognition of and calls to address the issues that contribute to the inequitable burden of chronic pain in society.^12,24,57^ The demonstration of how the COVID-19 pandemic has disproportionately affected patients with chronic pain living with low SES makes this review topical because it seeks to contribute to greater understanding of how access to chronic pain care can be improved for these patients.^58^ The information obtained from this review is relevant for clinicians, hospital administrators, and politicians with respect to becoming better informed of the barriers to accessing care for chronic pain. The results of this review may also be beneficial for establishing guidelines to deliver equitable pain management services to patients from differing socioeconomic backgrounds.

## Methodology

A scoping review was completed to assess the association of SES on physician prescribing patterns for adult patients experiencing chronic pain. The search strategy extracted articles that assessed the association of SES on physician prescribing practices for chronic pain management in adults. This review focused on studies addressing income and education given the importance of these determinants as previously described. Chronic pain was considered as “pain which has persisted beyond normal tissue healing time,” which, in the absence of other factors, is generally taken to be 3 months.^4^ We focused on studies that included chronic pain unrelated to cancer and studies that addressed common chronic pain conditions. We included studies related to arthritis, back/neck pain, headache disorders, fibromyalgia, and myofascial pain syndrome.

An electronic search strategy was established in collaboration with an information specialist librarian. The searches completed for this review were restricted to those in the English language and published between the years 2000 and 2021. The electronic databases used for the searches included EMBASE, CINAHL, SCOPUS, and Ovid MEDLINE. The key words related to SES used in the search included “social class,” “social status,” “education,” and “income.” The search terms related to prescribing practices included “physician prescribing practices,” “practice patterns,” and “inappropriate prescribing.” The search terms related to chronic pain that were used for this literature search were as follows: “chronic pain,” “persistent pain,” “arthritis,” “back pain,” “neck pain,” “headache disorders,” “fibromyalgia,” and “myofascial pain syndromes.” The search strategy was conducted to a final date of February 1, 2021, and was continually updated throughout the completion of this review. The search strategy identified 3492 articles to be included in the title and abstract screening for the scoping review. Any duplicate records were removed prior to screening.

The overarching goal of the scoping review selection was to identify and review any available evidence within the eligibility boundaries previously outlined. Specific screening questions were developed for all levels of the assessment and two independent reviewers performed the screening process. Two levels of screening were completed to identify the relevant studies for this scoping review. The first level of screening included a title and abstract review for the articles retrieved from the electronic search in order to remove any studies that did not relate to the review topic. The reports obtained from this screening process were uploaded to Covidence (www.covidence.org) to assist with the overall management of the articles for the review. The articles identified by either one or both reviewers as possibly relevant were retrieved for a full-text review. The second level of screening involved a full-text review based on the eligibility criteria previously outlined. A full paper analysis was completed to ensure that the study provided sufficient information relevant to the objectives of this scoping review. Following the second level of screening, the reference lists of the relevant articles were also manually searched to identify additional references. Disagreements arising between the two independent reviewers during the screening process were resolved by consensus. Thematic analysis of the selected studies was performed independently by each author and then reviewed together to develop consensus. This was an iterative process.

## Results

**Figure 1.**
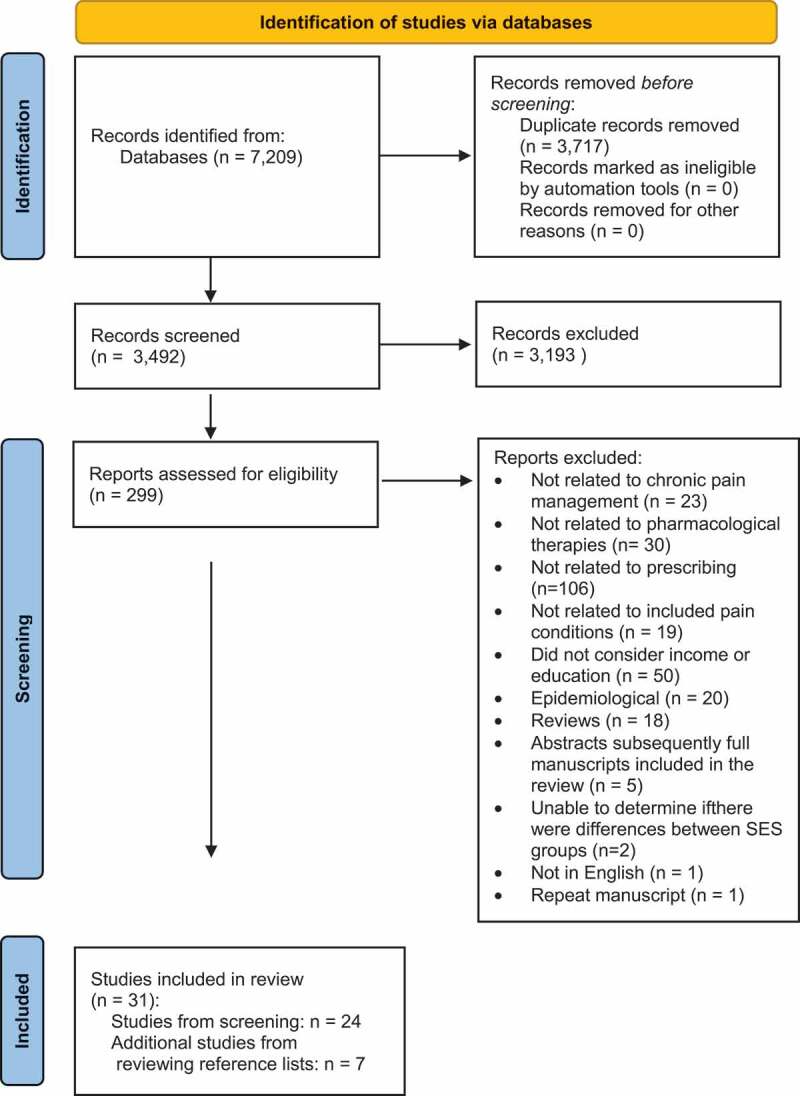
Article management search process.

During the primary review, 299 articles were selected to be reviewed further based upon their title and abstract. The full texts of these selected articles were reviewed and 24 papers met the inclusion criteria for this review,^59–82^ along with an additional 7 studies found after reviewing the reference lists from those studies (Table 1).^83–89^ Reasons for exclusion included that the studies (1) did not cover pain management,^90–112^ (2) did not deal with pharmacological therapies,^29,113–141^ (3) were not related to prescribing,^142–247^ (4) were not related to the pain conditions outlined in the inclusion criteria,^248–266^ (5) did not consider either income or education,^7,11,27,267–313^ (6) were epidemiological,^314–333^ (7) were reviews,^23,334–350^ (8) were abstracts that were subsequently published as full-length manuscripts that appeared in the search,^351–355^ (9) did not provide enough information to determine whether there were differences between SES groups,^356,357^ (10) were repeated,^11^ or (11) were not in English.^358^ A PRISMA (Preferred Reporting Items for Systematic Reviews and Meta-Analyses) flow diagram summarizes the article management process used for this review (Figure 1).^359^

**Table 1.** List of reviewed studies.

Study	Design	*n*	Condition	Treatment studied	Impact on prescribing pattern
Anastas et al.^59^	Observational cohort	436	Low back pain	Opioids	Physicians were more likely to recommend opioids for patients with lower SES
Bankole^60^	Retrospective cohort	116	RA	DMARDs	There was no relationship observed between
					SES and medications prescribed
Baser et al.^61^	Retrospective cohort	Not given	RA	IV vs SC therapy	Patients with higher SES were more likely to be prescribed an IV therapy
Bigal et al.^62^	Longitudinal questionnaire	6865	Migraines	Triptans	Low household income and lack of insurance were negatively associated with new triptan use
Celentano et al.^83^	Cross-sectional questionnaire	20,468	Migraines	Prescription medications	Differences seen in prescription medication use across income levels were not significant
Chu et al.^84^	Longitudinal questionnaire	11,388	Migraines	Triptans	Low household income, lack of insurance, and lower levels of education were negatively associated with new triptan use
Chuang et al.^63^	Cross-sectional observational	765	Neck, back, or osteoarthritis pain	Opioids	Patients who received Social Security income were more likely to receive opioids
Codreanu et al.^64^	Cross-sectional	4507	RA	bDMARDs	Lower or poor county socioeconomic indicators are associated with lower access to bDMARDs
Fanciullo et al.^65^	Survey	25,479	Spinal and radicular	Opioids	Level of education was not found to be related to the likelihood of being prescribed an opioid but patients prescribed opioids were more likely to be unemployed
			Pain		
Frenk et al.^85^	Survey	Not indicated	Pain	Opioids	Use of opioids decreased with increasing income
Frisell et al.^66^	Retrospective cohort	9310	RA	TNFi	Patients starting TNFi were more likely to have higher SES
Grol-Prokopczyk^67^	Survey	3721	Chronic pain	Opioids	Low wealth was a predictor of opioid use
Kuo et al.^68^	Cross-sectional observational	800,664	Chronic pain	Opioids	Low SES and residence in a lower education area were associated with a higher likelihood of receiving opioids
Lacaille et al.^86^	Cross-sectional observational	27,710	RA	DMARDs	Patients with lower SES were less likely to receive DMARDs
Lipton et al.^69^	Retrospective cohort	775	Migraines	Migraine therapies	Patients with higher SES were more likely to receive guideline-specified appropriate pharmacological management
McKeown et al.^70^	Retrospective cohort	1583	RA	DMARDs	Patients in community settings with higher SES were more likely to receive DMARDs
Nampiaparampil et al.^71^	Randomized controlled trial	90	Low back pain	Opioids	Interim analysis demonstrated that patients with lower SES were more likely to be prescribed opioids; this did not reach statistical significance in the final analysis
Nielsen et al.^72^	Retrospective cohort	1514	Chronic pain	Opioids	Having private health insurance facilitated access to nonopioid pain therapies. Higher opioid use was associated with increased financial and access barriers to nonopioid treatments
Nikiphorou et al.^73^	Cross-sectional observational	3984	Spondyloarthritis	DMARDs	Higher country welfare was associated with higher bDMARD use
Pensa et al.^74^	Observational cohort	76,432	Chronic pain	Opioids	Workers receiving hourly wages were more likely to receive opioids
Putrik et al.^75^	Retrospective cohort	1946	RA	bDMARDs	Lower level of education was associated with a reduced likelihood of being prescribed a bDMARD
Schmajuk et al.^87^	Retrospective cohort	93,143	RA	DMARDs	Those patients with the lowest ZIP code–based SES were the least likely to receive DMARDs
Shmagel et al.^76^	Survey	5103	Low back pain	Opioids	Lower education was associated with increased opioid use
Stokes et al.^77^	Cross-sectional	7256	Musculoskeletal conditions	Opioids	Reduced odds of being prescribed an opioid were observed for patients with lower levels of education
Stokes et al.^78^	Cross-sectional	4898	Arthritis or back pain	Opioids	Lower education and unemployed status were associated with greater opioid use
Tamayo-Sarver et al.^79^	Survey	5398	Migraines, back pain	Opioids	Having socially desirable characteristics (high prestige occupation) as a proxy for higher SES was associated with a greater propensity to prescribe opioids
Tatangelo et al.^80^	Retrospective cohort	17,672	RA	bDMARDs	Patients with lower SES were the least likely to receive bDMARDs
Wang et al.^81^	Retrospective cohort	875	Osteoarthritis	NSAIDs	Females with lower income were less likely to be prescribed newly enlisted NSAIDs
Wu et al.^88^	Longitudinal cross-sectional	1961	Migraines	Triptans	Patients with lower incomes or no insurance were less likely to receive triptan medications
Yazdany et al.^89^	Retrospective cohort	8125	RA	DMARDs	Patients with lower SES were more likely to be prescribed glucocorticoids alone rather than DMARDs
Yelin et al.^82^	Retrospective cohort	527	RA	DMARDs	Patients with a household income of <$30,000/year were less likely to receive DMARDs

Adapted from Page and colleagues.^359^

The results of the reviewed studies are summarized in Table 1. Overall, these studies demonstrated that SES and, more specifically, income and education typically had an association with physician prescribing patterns for chronic pain management. These associations can be categorized according to the association of SES on the provision of (1) opioid prescriptions, (2) medications to manage arthritis, or (3) triptan medications for headaches.

### Association of SES with Opioid Prescribing

Opioid prescriptions were the most common pharmacologic treatment studied among those reviewed. Most studies identified an association between chronic pain, lower SES, and a greater likelihood of receiving an opioid prescription.^59,63,65,67,68,72,74–78,85^ Looking at SES from an income perspective, studies found that individuals with lower income were more likely to be prescribed opioids for chronic pain management. For example, Grol-Prokopczyk^67^ identified that older American adults in the top income quartile had less than half the odds of using opioids to manage their pain compared to those in the bottom quartile. Frenk and colleagues^85^ looked at patients’ income from the perspective of where it related to the American federal poverty line threshold; those with family incomes less than 200% of the threshold were significantly more likely to use an opioid compared to those with incomes greater than 200% of the threshold. Others looked at the receipt of low-income subsidies, being paid hourly, or unemployment status as surrogate markers for low-income status and found that these were similarly associated with higher opioid prescription and use rates.^63,65,68,74,77,78^ In terms of doses of opioids prescribed, Nielsen and colleagues’^72^ cohort study of 1514 patients across Australia found that higher doses were provided to patients who had increased barriers, financial and access-wise, to nonopioid treatment. Instead of looking at data obtained from cohorts, cross-sectional surveys, or databases, Anastas and colleagues^59^ studied how physicians made pain management decisions using simulated patients with chronic back pain of varying SES backgrounds and also found that they were more likely to recommend opioid use for patients who had lower SES.

Looking at SES from an education perspective, patients with lower levels of education were found to have higher rates of opioid use.^76–78^ In looking at the management of 4898 American adults with chronic back or arthritis-related pain between 1999 and 2014, Stokes and colleagues^78^ observed that prescription opioid use was significantly greater for those without high school matriculation compared to those who had completed high school (22.7% versus 5.7%). These latter findings are consistent with those described by Shmagel and colleagues,^76^ who found that 94% of patients with chronic back pain to whom opioid prescriptions were provided had less than a college education.

The use of opioid prescriptions for those living with chronic pain in lower SES situations was found to further disadvantage them. Kuo and colleagues^68^ identified that individuals with a lower SES were more likely to utilize opioids to manage their chronic pain for a longer time, which resulted in an increased risk of opioid overdose–related emergency room visits and hospitalizations. In addition, Chuang and colleagues^63^ found that patients who used opioid analgesics to manage their chronic pain were more likely to be unable to work due to disability and were more likely to receive social security income. When controlling for potential confounders in multivariable analyses, patients who used opioid analgesics had three times the odds of being unable to work due to disability compared with nonusers.^63^

A limited number of the studies included in this review did not identify an association between SES and the likelihood of receiving an opioid prescription for chronic pain. For example, Nampiaparampil and colleagues^71^ did not find that opioid prescriptions were more likely to be prescribed to African American patients of low SES. Additionally, Tamayo-Sarver and colleagues^79^ identified that “desirable” patient characteristics increased the likelihood of receiving a prescription of opioids for chronic pain, by which they meant patients with higher SES, who were employed, and who had an established relationship with a primary care physician.^(p1239)^ Other studies found that lower education was found to be a weaker predictor of opioid use than income status^67^ or not predictive at all.^65^

### Association of SES with Prescriptions for Medications to Manage Arthritis

Several studies included in this scoping review identified a link between SES and the likelihood of receiving prescriptions of a disease-modifying antirheumatic drug (DMARD) for rheumatoid arthritis. Patients with greater financial resources were found to have a higher probability of being prescribed a biological DMARD.^64,70,73,80,82,86,87,89^ Patients with low personal incomes were more likely to be prescribed glucocorticoid monotherapy rather than a DMARD.^89^ Living in communities associated with lower SES was also found to be negatively associated with receipt of a DMARD prescription,^86,87^ and analyses demonstrated that this effect was evident even beyond the effect of individuals’ low personal income.^87^ Such a relationship was seen at even larger scales at the county and even national levels, even with the existence of prescribing guidelines.^64,73^ The time until initiation of a biological DMARD was also significantly longer for patients living in lower SES areas.^80^

Lower income was also associated with lower odds of being treated with newer or other medications for arthritis. Wang and colleagues^81^ gauged patients’ household income from income tax files and found that despite the presence of a universal national health care insurance system in Taiwan, patients were less likely to receive newly enlisted nonsteroidal anti-inflammatory drugs if they had lower incomes. The use of tumor necrosis factor inhibitors was also less likely for patients with lower SES.^66^

### Association of SES with Prescriptions to Treat Migraines

Studies analyzing the variability in triptan prescriptions for patients with migraines living in different socioeconomic situations mostly showed that lower SES was associated with a decreased propensity to receive them.^69^ Patients were more likely to use triptans if they had insurance coverage^62,84,88^ or higher incomes, which meant annual household incomes exceeding $60,000 compared to less than $22,500,^62^ exceeding $40,000 compared to less than $22,500,^84^ or high income levels compared to middle, lower, or poor income levels when income was referenced with respect to a federal poverty line threshold.^88^ Achievement of at least college-level education was also seen to be associated with an increased likelihood of using a triptan medication.^84,88^ It was only an American national stratified sample survey of households using a mailed questionnaire that found that income levels were associated with insignificant differences in rates of prescription medication use for migraine headaches.^83^

## Discussion

This scoping review looked at the association of SES on physician prescribing patterns for adult patients living with chronic pain. Chronic pain is recognized to be affected by a variety of biological, psychological, and social factors and therefore patients’ socioeconomic circumstances affect their pain and pain management experiences.^6,14,23,48,360,361^ Indeed, as Karran and colleagues^339^ wrote, “Health outcomes are explained less by the quality and availability of medical care than they are by the context and circumstances of people’s lives.^(p2476)^ Further, Brady and colleagues^7^ noted that “as pain represents higher-order processing, a product of individual interpretation and meaning, it is important that research into pain seeks to understand the ethnocultural and socioenvironmental contexts that shape the way people live with and manage pain.”^(p435)^

A scoping review was selected because the aim was to synthesize the evidence available regarding the associations of SES with specific chronic pain conditions and identify any knowledge gaps. It is recognized that SES comprises more than just one construct and so factors such as income and education were considered because these are thought to be valid and stable constructs.^6,362,363^ An approach was utilized to maximize the identification of all relevant studies associated with arthritis, back/neck pain, headache disorders, fibromyalgia, and myofascial pain syndrome, income and education, and prescribing patterns. Most of the studies included in this review utilized data collected from a variety of clinical settings. Many of the included studies had large cohorts of patients across different geographic locations, such as the United States,^59–63,65,67–69,71,74,76–79,82–89^ Canada,^70,80,86^ Australia,^72^ Norway,^75^ Romania,^64^ Sweden,^66^ Taiwan,^81^ and a multinational trial.^73^ This increases the generalizability of the findings but also means that the results need to be interpreted in the context of the heterogeneity of the health care systems in which the patients were studied. For example, some studies were performed in countries with universal health care coverage, whereas others were performed in systems in which patients bear more financial responsibility for their health care and prescription medications. Even countries with national universal health care systems may not have universal prescription medication coverage, as is the case in Canada, which means that patients with lower income may face challenges affording medications for pain management.

### Medications Prescribed

A variety of hypotheses have been suggested to explain the observed relationships between SES and opioid prescribing and use rates. It was thought that lower SES was associated with decreased resources and therefore abilities to access nonopioid therapies.^71^ This was due to the costs associated with other types of treatment as well as challenges with taking time to access other services.^77,78,364^ Nielsen and colleagues^72^ found that having private insurance facilitated access to nonopioid therapies as well as access to specialists. They additionally found that the use of higher doses of opioids was associated with greater challenges in getting to pharmacies and physicians, including specialists. As has been pointed out, the lack of coverage for a variety of nonopioid pain therapies therefore means that patients with lower SES may be unable to access more “widely recommended treatment options.”^76(p1110),365^ Prescribers’ recognition of this may thus lead them to overrely on opioids for pain management for patients with lower SES. At the same time, Grol-Prokopczyk^67^ suggested that caution is required as efforts are made to address the opioid crisis and the “crisis of undertreated pain” by limiting access to opioids: “To shut the door to one form of pain treatment without ensuring that others are open will likely exacerbate the latter crisis, and perhaps the former too, if those unable to access prescribed drugs switch to illicit ones.”^(p1017)^

From an education perspective, it has been suggested that patients with lower levels of education may have been found to be more likely to use or be prescribed opioids because they were not as well-informed about their lower efficacy in pain management and their associated side effects.^76^ There is evidence to support this assertion because taking into account health literacy when educating patients about opioid use for pain was an effective way of changing patients’ perceptions about their pain.^76,366–368^ The contrary finding by a few studies that higher SES was associated with greater rates of opioid prescribing was suggested to be potentially explained by physicians’ concerns that patients with lower SES would be less likely to follow their prescribing and treatment recommendations and more likely to misuse opioids.^59,71,345,369,370^

The association of lower access to DMARDs among those with lower SES was thought to be due in part to the cost of those medications. Lower incomes were thought to preclude the ability for patients to pay for them.^82,87,89^ This was thought to be in the context of either lacking or having limits to their health care insurance.^82^ Nevertheless, this relationship has been observed even when patients have access to universal health care insurance.^80,86^ Putrik and colleagues^75^ found that within such a health care system context, it was also patients’ decreased educational background that contributed to the lower likelihood of receiving a DMARD prescription. Having higher education was hypothesized to enable patients to gain better information about DMARDs and afford them better abilities to negotiate with their physicians about receiving DMARDs.^75^

The finding that even living in an area associated with lower SES was associated with reduced access to DMARDS suggests that less household or neighborhood income may have served as a proxy for access to specialist care, which was thought to improve patients’ ability to access DMARDs.^79,89^ Thus, it has been found that patients with lower SES were less likely to see rheumatologists.^80,82,89^ In the study by Yelin and colleagues,^82^ patients with lower SES were suggested to have access to federally run health centers that may have had fewer medical resources available to them. Moreover, physicians in those areas may have had less ability to access specialty care, like that provided by rheumatologists.^82^

The cost of triptan medications was thought to explain the observed association of their reduced prescription and use among patients with lower SES and/or lack of insurance coverage. Wu and colleagues^88^ noted that these medications can be costly, with a single triptan pill costing up to $46 in the United States, for example. Bigal and colleagues^62^ wondered whether patients’ access to universal health coverage would mitigate the observed decrease in triptan use for migraines because patients’ income would potentially be a less important factor affecting its use. Socioeconomic factors like income and education have been suggested to be surrogate markers for patients’ access to care, and this may explain the observed relationship between lower SES and reduced triptan use.^83,84^ It was suggested that strategies to improve migraine management for patients without insurance coverage for medication or with lower SES include increasing their use of prophylactic medications rather than triptans and enhancing those patients’ pain self-management skills.^88^

### SES Measures Studied

Income was measured in a variety of ways in the reviewed studies. In some studies, patients’ income was measured and then grouped into categories.^62,81^ Other studies looked instead at household income; Stokes and colleagues^77,78^ additionally looked at whether households received other types of income support, such as housing support, food stamps, child support, and unemployment insurance. Wang and colleagues^81^ adjusted for household structure because they argued that such structure influences what resources are available to individual members of the household. A marginalization index was used in one study in order to gather information related to domains such as residential instability, material deprivation, dependency, and ethnicity.^80^ Alternatively, income was expressed in terms of how it related to federal poverty limits.^88,89^ Rather than using patient-generated data, some studies relied upon the use of census data in order to determine neighborhood income quintiles.^76^ It has been argued that a drawback to this is that such estimations may not reflect the true income for individuals in the study.^81^

Access to health care insurance was another surrogate measure of income that was employed by some studies because it was thought that this would have implications for patients’ abilities to access prescription medications. Indeed, studies confirmed this association. For example, Nielsen and colleagues^72^ found that the greatest predictor of patients’ use of nonopioid pain management modalities was having private health insurance. They argued that subsidization of such treatments is therefore important because patients may use medications such as opioids out of necessity rather than choice.^67,72^ Increased wealth that affords the ability of patients to pay for a greater variety of pain management options also is argued to facilitate other advantages, such as the ability to work fewer hours, or may be associated with occupations that support workplace accommodations or protect against the worsening or progression of chronic pain.^14^ Patients with lower SES may also experience greater barriers to accessing insurance coverage that extend beyond payment alone, such as navigating bureaucracies.^24^ Even with universal health care coverage systems or government-sponsored insurance programs, patients with lower SES were found to have decreased access to certain medication therapies; in some cases this was thought to be due to such systems as still not providing adequate coverage for therapies.^72,79,82^ As Booher^23^ pointed out, patients with lower SES “become caught in the vicious cycle of chronic pain leading to disability and poverty, and poverty worsening chronic pain.”^371(p386)^

There was heterogeneity as well in the way in which level of education was measured in the reviewed studies. In some studies, it was viewed in a dichotomous manner looking at whether patients had less than or more than a college education or stratified in terms of the highest level of education obtained.^7–78,88^ Others looked at census data to obtain levels of education for ZIP code areas and used that information rather than details for individual patients.^68^ Putrik and colleagues^75^ suggested that a better way of looking at education would be to look at patients’ health literacy rather than looking at the level of education they had obtained because this might be more meaningful in terms of determining how or why level of education is associated with particular prescribing patterns.

### Prescribing Biases

The social context in which chronic pain clinical encounters take place as well as both implicit and explicit biases held by physicians can influence prescribing patterns and lead to disparities in pain management.^15,59,75,372,373^ Green and colleagues^15^ noted that decisions about pain management are affected by characteristics of patients, health care providers, the clinical practice environment, and the overall health care system in which pain care is provided. Physicians may “unconsciously consider judgements beyond clinical factors in their decisional processes.”^75(p1222)^ This is especially true for conditions like chronic pain, argued Anastas and colleagues,^59^ because of pain’s “subjective nature” and “clinical uncertainty.”^(p772)^ As they put it, “Providers’ attitudes may ‘fill in the gaps’ of insufficient or ambiguous information, resulting in systematic differences in chronic pain care across race and SES groups.”^59^^(p772)^

There is extensive literature demonstrating bias in how health care providers treat patients from specific ethnocultural backgrounds.^15,280,374–377^ For example, compared to the experience of White patients, Black patients’ pain has been shown to not be as aggressively assessed or treated, they are less likely to be referred to pain specialists,^15,280,374,375,377^ and they are more likely to be required to provide urine drug screens.^376^

Tamayo-Sarver and colleagues^79^ suggested that inequities in the delivery of pain care may not be due to ethnocultural status alone and that SES is implicated as well. Less common are such studies looking at bias in how patients with lower SES have been treated,^59,79^ but the studies that have been done that specifically look at the way in which variability in health care provider and patient characteristics affects chronic pain management decision making have confirmed that lower SES can lead to inequities in pain care.^71,378,379^ Patients with lower SES and chronic pain have been viewed by health care providers to be less competent and compliant in the use of medications and other multidisciplinary therapies for pain.^163,369^ They have been thought by physicians to have less self-control over medication use with a greater propensity to misuse opioids and so studies have found that physicians disproportionately require them to complete opioid contracts compared to patients with higher SES.^59,273,369,380^ Patients with lower SES have been considered by health care providers to be more “demanding,”^163(p2094)^ and their pain has been referred to in “dehumanizing” ways.^378(p152)^ Tamayo-Sarver and colleagues^79^ wrote that SES was associated with “physicians’ perceptions of patients’ abilities, personalities, behaviors, and role demands” and that physicians “may treat patients preferentially if there is less social distance between them.”^(p1245)^ Green and colleagues^15^ suggested that more research is needed to better understand the factors that “systematically influence” physicians’ decisions regarding pain management.^(p286)^

Characteristics of the communities in which patients reside also affect their ability to access prescription medications for chronic pain. Residing in communities with lower SES has been found to cause increased exposure to violence, psychosocial distress, and social isolation and, thus, to increased risk factors for the development and furtherance of chronic pain.^23,166,363,381,382^ Poorer communities are less likely to be situated in proximity to pain clinics, making it more challenging for their residents to access them.^302,383,384^ Moreover, access to medications in communities associated with lower SES has been found to be an issue because pharmacies in those areas may not have sufficient supplies of medications compared to pharmacies located in communities with higher SES.^294,382^ Morrison and colleagues^385^ found that disparities in supply were seen when looking from an ethnocultural perspective as well. Even after adjusting for rates of crime, pharmacies in New York City in Hispanic and African American neighborhoods were less likely to supply opioids compared to those located in White American ones.^385^

Physician prescribing practices for chronic pain management are influenced by practice guidelines. In their study including in this review, Stokes and colleagues^77^ identified that there was a decline in the number of individuals receiving opioids during the years 2013 and 2016 and suggested that this may reflect changes in physician prescribing practices as awareness of the opioid epidemic grew and as Centers for Disease Control and Prevention guidelines about reducing opioid use were developed. Nevertheless, they still found that there was an association between SES and the likelihood of being prescribed opioids for chronic pain management.^77^

### Socioeconomic Status and Intersectionality

In this scoping review, SES was looked at from the perspective of income and education but there are other components of SES that could be investigated. Similar relationships between SES factors such as level of education and income and chronic pain have been found for ethnicity. As Fuentes and colleagues^363^ noted, “Race and socioeconomics are entangled constructs sometimes interacting and sometimes acting individually”^(p1160)^ and thus they argued that SES and ethnicity cannot be used interchangeably. Patients considered to be ethnic minorities in North America have been found to have a disproportionate burden of chronic pain and lack of access to pain management.^382^ For example, African Americans have been found to have increased chronic pain prevalence, intensity, and pain-related distress, disability, and interference of function compared to other ethnic groups.^20,280,371,379,382,386–388^ Moreover, they have been found to have increased exposures to risk factors for the development of chronic pain and to be offered less comprehensive pain management options.^345,382^ The etiology of this is multifaceted, including mistrust of the health care system at the individual level^363^; decreased access to health care resources such as therapies, primary care physicians, and pain specialists at the health system level^345,382^; and negative stereotypes and discrimination at the societal level.^273,377,380,382,389^ It has therefore been suggested that research related to the effects of SES on access to pain care should also consider patients’ ethnocultural status and recognize that socioeconomic advantages and disadvantages are not uniform across ethnocultural groups.^28,59,361^ As Riskowski^28^ pointed out, patients’ income, education, social status, and ethnicity may each contribute in some way to “the development of pain or in the management of pain, or lack thereof … these factors together can create access to vital, influential, and extensive resources and opportunities to impact health and health behaviors.”^(p1517)^

Brady and colleagues^7^ pointed to Crenshaw’s^390^ theory of intersectionality as providing a useful way of depicting the manner in which the variety of factors that contribute to patients’ social circumstances and identities interact to affect their experiences with chronic pain. They described that patients’ life experiences and identities “interact to position an individual along a specific trajectory that may confer a position of advantage or disadvantage for health” and “can factor into the creation and maintenance of chronic pain disparities.”^7(p435)^ Bonathan and colleagues^14^ outlined in particular how lower SES and chronic pain are interconnected:

The consequences of feeling socially inferior, living in a less socially cohesive neighborhood with a more imminent sense of threat, combined with poorer education and, therefore, poorer job opportunities are likely to interact with the psychological factors known to increase the risk of chronic pain … financial restrictions also constrain coping strategies and since some coping is consistently associated with greater disability, this is a self-maintaining process. Educational level may also influence choice of strategies.^(p161)^

Therefore, to better understand why chronic pain is not addressed in the same manner for all patients, an understanding of the “conditions in which people are born, grow, live, work, and age, and the inequities in power, money, and resources that are responsible for disparities in health outcomes” is necessary.^339(p2476)^

### Limitations

The results of this review must consider the limitations associated with the reviewed studies. First, there was heterogeneity among the studies from several perspectives. As noted earlier, studies used different ways of measuring SES, in terms of both income and education. Studies used various methodologies; some used data from administrative databases, which can be associated with concerns about the accuracy of diagnoses used and thus the manner in which cases were selected.^86^ Others used cross-sectional surveys that often use patient self-report in order to acquire date. Such studies are therefore associated with potential recall bias and reporting inaccuracies.^62,76^ Information may not have been verified by review of charts or provider information.^84^ Different studies adjusted for different variables when looking at pain-related outcomes. For example, Chuang and colleagues^63^ controlled for potential confounders including depression, pain severity, pain interference, global physical and mental functioning, and demographic characteristics, whereas Grol Prokopczyk^67^ controlled for age, sex, race/ethnicity, and marital status. Second, the retrospective nature of most of the reviewed studies meant that they could only show associations and could not determine causality.^63^ Third, studies that looked at prescribing patterns may be inaccurate, because reports based upon self-report may not reflect what physicians recommended. Additionally, the provision of a prescription does not necessarily mean that it was filled^67^ or that medications were actually taken by patients.^68^ There may have been reasons why patients were not provided with certain treatment options, and this was often not reported in studies (contraindications, used in the past and failed, or had adverse events to a particular pharmacological agent).^67^ Fourth, various elements of SES were at times grouped together. For example, Nampiaparampil and colleagues^71^ grouped lower SES related to income and education together with ethnicity and so this would make distinguishing the effects of ethnicity itself from SES challenging.

There are limitations associated with this scoping review as well. Booher^23^ used key words such as “socioeconomic status” and “chronic pain” but noted that this may have led to missing some studies; this limitation applies to this scoping review as well. A variety of databases that include publications related to medicine, nursing, public health, sociology, and pain were used in order to ensure that as many relevant studies were found as possible. However, limiting the search to particular databases may have led to missing studies. Additionally, the factors that lead to a patient being prescribed a specific medication for chronic pain management are known to be complex and involve a variety of steps.^84^ For example, patients must be able to access a health care provider, receive a diagnosis, receive a prescription, fill that prescription, and then take that medication.^84^ There are factors associated with SES that can influence each of these steps. This scoping review’s focus on patients being prescribed or taking a medication therefore misses some of these other factors. Not all types of chronic pain conditions were considered in this scoping review, and so its findings cannot be generalized to chronic pain conditions beyond those described in the inclusion criteria. A final limitation is that this review only includes articles that were published in English. Despite these limitations, we identified a relatively consistent pattern between SES (specifically, income and education) and its association with chronic pain management using prescription medications.

## Conclusions

This scoping review provides insight into the complex interactions between SES, specifically income and education, and access to pharmacological treatments for specific chronic pain conditions. The studies examining this topic are heterogenous in terms of how they considered income and education, the methods they used, and the conditions and medications they studied. Nevertheless, an association was found such that patients with lower SES typically do not receive equal prescription medicine opportunities for their chronic pain conditions.

Further research is required to better understand hpw lower SES is associated with disparities in the provision of pharmacological pain management with the aim of reducing the magnitude of such disparities. Green and colleagues’^15^ recommendations about how to address the gap in knowledge related to ethnicity and pain care can be applied in considerations of how to address this gap related to SES. At the level of the patient, more knowledge is needed from the perspective of patients with chronic pain and lower SES regarding their needs, the challenges they experience obtaining pain management related to their income and education status, and their goals and expectations for treatment. Although this review focused on income and education as they pertain to SES, other areas that require attention are the disparities in access and care that patients receive based upon other factors, such as gender, ethnicity, and language. At the provider level, more research is needed to understand how patients’ income and education affect physicians’ decisions about chronic pain management and about stereotyping and biases that may be involved. At a broader level, more research has been recommended to understand how patients’ income and education intersect with various other social determinants of health to create, influence, or exacerbate chronic pain management disparities.

Addressing disparities in chronic pain care will similarly require a multitiered approach, with particular strategies at the level of the health care provider. With regard to addressing issues related to patients’ education, this could involve improving provider education concerning patients’ health literacy.^391^ Health care providers have been found to overestimate patients’ understanding of clinical information.^391,392^ It has been suggested that providers use literacy-dependent teaching methods to provide information using a variety of modalities.^393–396^ This would help promote shared clinical decision making.^393^ With regard to addressing issues related to patients’ income, this could involve health care providers having open discussions with patients about the costs of pharmacological therapies.^397^ Indeed, there is evidence to suggest that many providers consider it their responsibility to have such conversations with patients.^398,399^ Among the suggestions that Kolhatkar and colleagues^397^ have for prescribers to improve medication affordability are their “staying up to date on drug costs, prescribing the most cost-effective alternative, frequently reviewing medication prescription regimens for opportunities to deprescribe and prescribing generic drugs.”^(pE549)^ It is important that health care providers be vigilant in considering the various factors that influence their prescribing practices. Physicians should be encouraged to examine their own implicit assumptions and behaviors toward chronic pain.^59^ Moreover, it has been suggested that clinicians consider as well the financial conflicts of interest that may influence their prescribing of more expensive medications.^400^ In accordance with the recommendations of the Canadian Pain Task Force,^12^ other prescriber-level strategies include improving health care provider education of chronic pain as well as ensuring that its assessment occurs within a biopsychosocial framework that considers patients’ social determinants of health. Using a biopsychosocial approach to chronic pain conditions may assist in addressing the inequitable prescribing patterns among physicians based on a patient’s socioeconomic status because it would involve understanding the association of the patients’ pain with different aspects of their lives and vice versa. Understanding of patients’ socioeconomic situations can help better identify care options that are appropriate for them.

Higher-level strategies could focus on health care system reforms to improve patients’ access to evidence-based pharmacological, physical, psychological, and spiritual treatments for chronic pain.^14^ First, expansion of insurance coverage for pain therapies is required. Programs in the United States such as Medicaid and those provided by the Veterans Health Administration have been shown to significantly improve patients’ access to pharmacological therapies and increase medication treatment adherence.^401–404^ It has been suggested that further reducing or eliminating any co-payments that patients need to provide could further improve access to care for patients with lower SES.^405,406^ In Canada, the Canadian Pain Task Force^12^ recommends the expanding of patients’ access to pain treatments under provincial health care coverage and working with private insurers to incentivize expanded coverage of pain management options. Some provincial governments have endorsed improving patients access to currently non-insured nonpharmacological therapies for chronic pain.^407^ Previous studies have demonstrated that universal access to health insurance is not adequate to achieve total equity in access to appropriate pain management treatments.^72,75,80,81,86^ Therefore, it has been suggested that programs of universal coverage of prescription medications focused on equity should be developed to ensure that medications can be provided to patients based upon their needs.^408,409^ Second, the creation of national clinical guidelines to facilitate “evidence-informed, trauma-informed, equity-oriented, and biopsychosocial management of chronic pain across health care settings”^12(p10)^ has been recommended. This sort of advocacy has been done by clinical organizations with the assistance of experts in health policy, pharmacoeconomics, behavioral sciences, clinical care, and the pharmaceutical industry in order to support the development of national policies to increase the affordability of medications.^410^ The importance of such health system reforms is made clear, as Brady and colleagues^7^ noted that “recognizing chronic pain not only as a physical, socioeconomic or psychological problem but as an interaction between social class, migration class, gender, ethnoculture, and the health system encounter would facilitate framing of health disparities as a function of the system, rather than a responsibility of … specific communit[ies].”^(p442)^
